# Healthcare Providers’ Perception and Barriers Concerning the Use of Telehealth Applications in Saudi Arabia: A Cross-Sectional Study

**DOI:** 10.3390/healthcare10081527

**Published:** 2022-08-13

**Authors:** Saeed M. Alghamdi, Abdulelah M. Aldhahir, Jaber S. Alqahtani, Rayan A. Siraj, Abdullah S. Alsulayyim, Abdullah A. Almojaibel, Munyra Alhotye, Abdullah M. Alanazi, Abdullah A. Alqarni

**Affiliations:** 1 Clinical Technology Department, Respiratory Care Program, Faculty of Applied Medical Sciences, Umm Al-Qura University, Makkah 21961, Saudi Arabia; 2 National Heart and Lung Institute, Imperial College London, London SW3 6LR, UK; 3 Respiratory Therapy Department, Faculty of Applied Medical Sciences, Jazan University, Jazan 45142, Saudi Arabia; 4 Department of Respiratory Care, Prince Sultan Military College of Health Sciences, Dammam 34313, Saudi Arabia; 5 Department of Respiratory Therapy, College of Applied Medical Sciences, King Faisal University, Al-Hasa 31983, Saudi Arabia; 6 Respiratory Care Department, College of Applied Medical Sciences, Imam Abdulrahman Bin Faisal University, Dammam 31441, Saudi Arabia; 7 Department of Respiratory Therapy, College of Applied Medical Sciences, King Saud Bin Abdulaziz University for Health Sciences, Riyadh 12271, Saudi Arabia; 8 King Abdullah International Medical Research Center, Riyadh 11481, Saudi Arabia; 9 Department of Respiratory Therapy, Faculty of Medical Rehabilitation Sciences, King Abdulaziz University, Jeddah 22254, Saudi Arabia

**Keywords:** telehealth, healthcare providers, barriers, perception

## Abstract

Background: Telehealth services are widely used in Saudi Arabia. Despite this, neither the use rate nor the attitudes, perceptions, and barriers concerning telehealth applications have been evaluated nationally from the perspective of healthcare providers (HCPs). Aim: This study aims to explore the use rate of telehealth, as well as the attitudes, perceptions, and barriers concerning telehealth use in Saudi Arabia from the perspective of HCPs. Methods and design: A cross-sectional survey was conducted and distributed to all HCPs between 16 November 2021 and 16 March 2022, through an online platform (Survey Monkey). Results: Overall, 1034 HCPs completed the online survey, of which 65.0% (*n* = 677) were male. Physicians accounted for 22.34%, while nurses and respiratory therapists accounted for 22.34% and 21.47%, respectively. Only 491 HCPs (47%) have used telehealth applications, the majority for less than a year (21.47%) or from one to three years (14.51%). Around 44% of HCPs perceived telehealth as being useful in quality and care delivery. Around 43% of HCPs felt comfortable using telehealth, and 45.45% perceived telehealth as being useful for patients with transportation difficulties. Additionally, 38% believed that telehealth provides a confidential way of protecting patients’ information, and 36% would like to receive more training in telehealth. Speech-language therapists and public health professionals were the highest HCP users (98% and 95%, respectively), while general physicians and dentists were the lowest users (44% and 55%, respectively). Lack of time or a busy schedule was the most common barrier to not using telehealth among all HCPs (38%). Conclusion: The use of telehealth was perceived as being positive as well as valuable and confidential in monitoring and providing care. However, challenges such as the lack of time or a busy schedule impeded the use of telehealth among HCPs in Saudi Arabia.

## 1. Introduction

Telehealth refers to a healthcare service that can be carried out remotely, irrespective of both the patient’s and the HCP’s geographic location [1]. There are various interchangeable terms used to refer to telehealth applications in clinical settings, such as telemedicine [2]. Globally, the application of telehealth approaches in clinical practice is increasing, as it has been shown that telehealth reduces healthcare costs and improves the quality of care [3]. In Saudi Arabia, there have been numerous advancements in telehealth. In 2017, the Ministry of Health (MOH) launched its new strategy, aiming to improve the quality and efficiency of healthcare services and to maximize accessibility among patients and HCPs irrespective of their geographic locations [4]. Between 2018 and 2020, several health applications such as Seha, Tawakkalna, Mawid, Tabaud, and Tataman were introduced by the MOH to improve the provision of telehealth in Saudi Arabia [5,6]. Published reports show that currently, there are more than two million users for telehealth services supported or provided by the MOH in Saudi Arabia [2].

Despite the high demand for telehealth services in clinical practice, several barriers and challenges in the use of telehealth applications have been identified from the perspectives of the public and HCPs [7]. In 2012, Azza et al. conducted a study to identify the willingness and perceptions of HCPs of using digital health in the Eastern Province of Saudi Arabia [7]. In addition to the lack of sufficient knowledge about other services and the benefits of telehealth, difficulties in the application of telehealth and a lack of time to adopt telehealth have been identified as common barriers to not using telehealth [7]. In 2020, the World Health Organization (WHO) declared the COVID-19 disease outbreak a pandemic [8]. The emergence of the coronavirus disease led to a dramatic increase in the use of digital health in Saudi Arabia and overwhelmed healthcare workers and telehealth platforms [2]. As a result, several challenges in telehealth services have emerged. Recently, it has been reported that physicians from five different hospitals in the Riyadh region of Saudi Arabia still have little knowledge of telehealth and its use [9]. These findings also demonstrated that physicians believe that factors related to privacy, cost, and information/communication technology may limit the use of telehealth [9]. Moreover, Aldhahir et al. conducted a national study to evaluate the perception of using a telehealth app (Seha) from the perspective of the public, as well as common barriers to their use [10]. The telehealth app was perceived as being reliable and easy to use, while non-users reported that a lack of knowledge was the most common barrier to not using the telehealth app [10]. Our research group explored the use of telehealth from the perspective of the general population using real-time data. The study demonstrated that there were more than 23 million registered users in digital health platforms in Saudi Arabia. The rate of telehealth use increased dramatically over two years (the report covers the period from 2019 to 2021) [11,12].

Although there has been an exponential increase in the use of digital health in Saudi Arabia, the use rate of telehealth, as well as the attitudes, perceptions, and barriers toward using telehealth applications from the perspective of HCPs have not been evaluated nationally. Additionally, we still have inconclusive data for determining in which professions telehealth applications are feasible and applicable. Therefore, this study aims to determine the use rate of telehealth, as well as attitudes, perceptions, and barriers when using telehealth applications from the perspective of HCPs in Saudi Arabia.

## 2. Methods and Design

### 2.1. Study Design

A cross-sectional survey was conducted through an online platform (Survey Monkey) between 16 November 2021 and 16 March 2022.

### 2.2. Study Population

Convenience sampling techniques were used to recruit the study participants. The main targets of the study were HCPs; no specific profession constituted the inclusion criteria. To ensure countrywide sample representativeness, professional societies were asked to distribute the survey to registered members using their official platforms. Additionally, we contacted members of professional committees and professional groups on social and professional networks (LinkedIn, Twitter, WhatsApp, and Telegram).

### 2.3. Questionnaire Tool

The survey was originally developed and validated by Almojaibel et al. [13,14]. Before participants answered the questionnaire, they were informed of the purpose of the study, the estimated completion time, data confidentiality, and voluntary participation. Participation in the survey was voluntary, and participants could withdraw without consequences. An additional statement was provided in the survey: “By answering ‘yes’ in completing the survey questions, you voluntarily agree to participate in this study and give your consent to use your anonymous data for research purposes”. The expected completion time was approximately five minutes. The questionnaire consisted of seven sections. Section one requested demographic information, including age, gender, profession, years of clinical experience, geographical location, type of hospital, and telehealth use in general; the participants had two choices (yes/no). If the participant answered ‘yes’, the questionnaire proceeded to years using telehealth and the full questionnaire. If the participant answered ‘no’, they were redirected to the last question of the survey concerning barriers. Section two was the awareness domain, consisting of three statements to agree or disagree with. Section three was the attitude domain, consisting of three statements. Section four was the practice domain, consisting of seven statements. Section five was the beliefs domain, consisting of twelve statements. Section six was the training domain, consisting of four statements. From section two to section six, the answers of the participants were recorded using a 5-point Likert scale ranging from 1 (=strongly disagree) to 5 (=strongly agree). The last part of the questionnaire consisted of a question related to barriers and limitations concerning the use of telehealth applications.

### 2.4. Ethical Approval

Institutional Review Board approval for the study was obtained from the Ministry of Health (Al Noor Hospital, Makkah), ID number (H-02-K-076-0820-361).

### 2.5. Statistical Analysis

Data were analyzed using the Statistical Package for Social Sciences (SPSS software, Version 25, IBM, Armonk, NY, USA). Descriptive analysis was conducted to report continuous and categorical variables. Continuous variables were reported in terms of mean and standard deviation. Categorical variables were reported and presented in percentages and frequencies. Statistical significance was set at *p* < 0.05. We calculated the use rate of telehealth among participants who had experience in telehealth. We defined the use rate as the number of years using telehealth divided by the total amount of clinical experience in years, with the use rate expressed as a proportion [15,16,17].

## 3. Results

Overall, 1034 HCPs, 677 male (65%) and 357 females (35%), completed the online survey between 16 November 2021, and 16 March 2022. There were 18 professional specialties using telehealth applications in clinical settings. Physicians constituted 22.34%, followed by nurses (21.47%), respiratory therapists (17.99%), physiotherapists (11.61%), and other specialties such as pharmacists and radiology technicians (Table 1). The mean and SD of clinical experience for all HCPs was 6 (±5) years. Out of 1034, only 491 (47%) had used telehealth applications. We also categorized telehealth use into less than a year, one to three years, four to six years, seven to nine years, and more than/equal to ten years. Out of 491 (47%) HCPs who used telehealth, the majority had used telehealth for less than a year (21.47%) or from one to three years (14.51%).

### 3.1. Awareness Domain

Awareness of telehealth applications in clinical facilities was the first domain of the questionnaire. The study demonstrated a high level of awareness of telehealth applications in clinical settings. Around 44% of HCPs strongly agree that telehealth was useful in quality and healthcare delivery. Additionally, 39.95% mentioned that telehealth was easy to use, and 40.91% thought positively about the use of telehealth in clinical facilities (Table 2).

### 3.2. Attitude Domain

The second domain was the attitude domain. We found that 43.06% of HCPs felt comfortable using telehealth, and 39.23% of the participants had the intention of using telehealth when applicable and would recommend using telehealth to a patient in a clinical facility (39.23%).

### 3.3. Practice Domain

The third domain was the practice domain. Around 34% of the responders strongly agree that telehealth applications have changed their working routine, and 38.04% strongly agree that telehealth applications have helped to enable quick access to patient information. Under this domain, the response to the statement “everyone in my workplace uses telehealth” was 28.23% strongly agree, 26.32% agree, 24.16% neutral, 18.42% disagree, and 2.87% strongly disagree.

### 3.4. Beliefs Domain

The fourth domain was beliefs. Among the HCPs who used telehealth applications, 29.67% strongly agree that telehealth implied major modifications in their routine clinical practice, 30.86% strongly agree that telehealth was also a good facilitator for patient care, and 34.69% strongly agree that it is a good idea to use telehealth to monitor patients in the clinic. Additionally, 33.97% strongly agree that telehealth applications in their clinical settings are reliable, and 38.28% strongly agree that telehealth is a confidential tool for care delivery.

### 3.5. Training Domain

The fifth domain explored training among HCPs: 36.60% of the HCPs stated that they would like to receive more training on telehealth, and 35.65% felt comfortable training their peers to use telehealth within the clinical setting.

### 3.6. Use Rate Per Profession

Speech-language therapists and public health professionals were the most frequent users of telehealth (98% and 95%, respectively). General physicians and dentists were the least frequent users of telehealth (44% and 55%, respectively). The use rate for all professions is reported in Table 3.

### 3.7. Barriers

**Figure 1:**
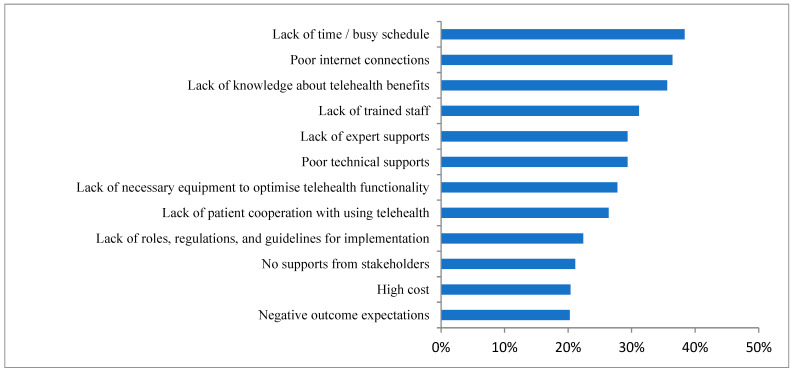
Barriers to Using Telehealth Applications among Healthcare Providers (*n* = 947).

A variety of barriers to not using telehealth in clinical settings were reported as follows: lack of time/busy schedule (38%) was the most frequently reported barrier, followed by poor internet connection (36%), lack of knowledge about telehealth (36%), and lack of trained staff (31%).Another reported barrier was a lack of expert support (29%). Barriers to using telehealth applications among healthcare providers are reported in Figure 1. When data were available, we reported barriers per profession as demonstrated in Supplementary Figures S1 and S2.

## 4. Discussion

To the best of our knowledge, this is the first national cross-sectional study exploring the perception of telehealth among HCPs and reporting on the use and barriers concerning telehealth applications among professionals in clinical settings in Saudi Arabia. Our main findings demonstrate that HCPs perceive telehealth as a useful, easy-to-use, effective method for monitoring, delivering, and following up on patient care. Additionally, HCPs recommend using telehealth, and feel confident about training patients or health-professional peers. Furthermore, HCPs are confident that patients’ data are transmitted reliably and confidentially; they feel comfortable; and they intend to use telehealth since it changes their working routine, implies positive modifications, improves practice, and allows for quick access to patient information. Moreover, HCPs believe that the workplace has sufficient technology and structure to support telehealth and perceive telehealth as a valuable tool for patients with transportation difficulties. Patients accept and like using telehealth. HCPs would like to receive more training in telehealth. Lack of time, knowledge, and trained staff, as well as poor internet connection are potential barriers to telehealth use.

This national survey reveals that awareness, knowledge, attitudes, and beliefs concerning telehealth are gradually spreading across Saudi Arabia’s healthcare professions. These results were as expected because medical institutions have accelerated the implementation of telehealth as an alternative method to providing care or monitoring patients, particularly after the emergence of the COVID-19 pandemic [10]. Another highlight of our findings is that several healthcare institutions in Saudi Arabia currently collaborate with the Saudi Central Board for Accreditation of Health Centers to establish an accreditation program (CBAHI) for remote healthcare services including telehealth [18,19]. More importantly, optimizing the accessibility and use of telehealth services across the Kingdom of Saudi Arabia is one of the targets of the Saudi Vision 2030 plan, which stresses the importance of adopting and developing a national telehealth network involving all healthcare professions [12]. These promising indicators optimized adopting telehealth solutions in healthcare professions, gaining more attention and support. Despite the emergence and exponential rise of telehealth in Saudi Arabia, there is still a need to promote awareness of the benefits of telehealth applications among different HCPs and decision-makers [20,21].

In the context of clinical practice, our results demonstrate that experience using telehealth makes routine work quicker. Additionally, HCPs felt confident in general using telehealth for their patients, the majority feeling that they had sufficient training in using telehealth in the clinical setting. Our findings are supported by previous studies showing that the effective use of telehealth in a clinical setting requires a high level of training and confidence [22,23,24]. Correlatively, a high level of training will require extra time and effort on the part of HCPs to use the telehealth applications effectively and safely [25,26]. However, lack of time was reported by all HCPs as the most widespread barrier to using telehealth.

Our results show that general physicians and dentists reported the lowest use rate in telehealth applications (44% and 55%, respectively). Limited use among general physicians could be related to the fact that telehealth applications may lead to misdiagnosis, and telehealth limits the patient–physician interaction (i.e., physical examinations) necessary for proper diagnosis [27,28]. Similarly, a cross-sectional study found that general physicians saw themselves as limited when practicing telehealth because of technical issues, including the availability of resources to allow for a complete, accurate diagnosis [29,30]. With respect to dentists, the low telehealth use rate could be related to what was published earlier regarding the low rate of using telehealth, i.e., the lack of awareness among dentists of teledentistry services in Saudi Arabia [31]. Similarly, other researchers in an exploratory study reported an urgent need to increase awareness of telemedicine among dental healthcare professionals [32]. It has also been reported that the use of and access to regular dental care are limited, and patients mostly visit the dentist for procedures [33,34].

In addition, our study reports that speech-language therapists and public health professionals use telehealth the most in their clinical practices (98% and 95%, respectively), perhaps due to the high demand for telehealth solutions among these professions [23,35]. Concerning speech-language therapy, therapies such as “singing for lung health” or “breathing exercise” are mostly provided using telehealth as they are flexible solutions [36,37]. Additionally, implementing these interventions via telehealth applications has been reported to have advantages over non-telehealth ones (85% versus 15%) as well as being effective in terms of maintaining adherence to the treatment, which may have a role in improving the patients’ clinical outcome [37,38]. Using digital technology in public health could facilitate preventive programs and help with more effective communication with the public and decision-makers, the same way that using data could facilitate more informed and efficient services even before the coronavirus pandemic.

Our study found that lack of time/busy schedule, knowledge, and training programs were the most widespread barriers preventing HCPs from using telehealth. In clinical practice, it has been reported that the use of telehealth applications relies mainly on the level of knowledge and skills, and to what extent this option is available and easy to access within the healthcare organization [39,40]. In addition, technophobia, the fear of unknown technology, is a potential factor that could negatively impact the use of telehealth in some clinical staff [41,42,43].

We also found that a poor internet connection is a major barrier from the perspective of HCPs. This is not surprising as technical problems are considered a common barrier associated with low-level use and satisfaction [10,21]. The uniqueness of our results, besides general perception, consisted of reporting barriers per profession (Supplementary Figures S1 and S2). This finding can help inform policymakers and stakeholders about the current barriers distributed in professional categories and how to mitigate them. Additionally, our results provide a current estimate of the use rate in each profession based on the available resources. It allows for comparing the feasibility of telehealth applications across HCPs over the years. In recent national reports about the digital transformation in the MOH, there was an increase in telehealth users in the current year. This report could be related to the MOH’s efforts to accelerate the digital health transformation and the empowerment of HCPs through developing and maintaining high-quality telehealth services [44]. However, the feasibility of telehealth applications across professions has many opportunities to develop a tailored service. This process may involve exploring the accessibility and real-time use (i.e., login data and time spent on software) on a large scale with the help of in-depth research methodologies such as qualitative research. It is necessary to investigate the actual use with machine learning and artificial intelligence to explore the patterns of use and reliability of telehealth in these professions.

However, our study shows a positive perception of telehealth among HCPs in clinical settings, similar to findings in previous studies suggesting that the level of awareness and knowledge of telehealth is gradually increasing [45,46,47]. The current literature covers mostly physicians, and there are limited data exploring perceptions in other healthcare professions [43,46,47,48].

### Limitations 

Caution should be exercised in describing the results from this national study as the survey was interrupted by the COVID-19 pandemic, which may have affected the perception of the actual use of telehealth technology. The pandemic has indeed driven the implementation of telehealth in most professions, but it is still not the normal situation for using and utilizing telehealth. Even though our sample covers most of the healthcare profession, we could not capture the response and perceptions of some HCPs regarding the use of telehealth. It was not possible to assess the perception of telehealth for those who do not have internet access or telehealth access. It would be better for future studies to find the actual use of telehealth by tracking real-time data. Altogether, the findings of this study should be interpreted with caution; the survey should serve as a baseline for future work on evaluating the use of telehealth in healthcare professions.

## 5. Conclusions

HCPs utilize telehealth services or applications in Saudi Arabia. HCPs perceive telehealth as being beneficial in improving care and facilitating care delivery to patients. However, current widespread barriers must be addressed to increase the awareness and the rate of using telehealth in some health professions in the future.

## Figures and Tables

**Table 1:** Demographic data and characteristics of all study respondents.

Variable	Frequency (%), Median [IQR], and M ± SD
**Age**	44 [28 to 59]
**Gender**	
Male	677 (65.0%)
Female	357 (35.0%)
**Location**	
Western Region	414 (40%)
Central Region	187 (18%)
Southern Region	175 (17%)
Eastern Region	155 (15%)
Northern Region	103 (10%)
**Type of hospital**	
Primary care	497 (48%)
Tertiary hospitals	537 (52%)
**Have you used any form of telehealth application in your workplace**	
Yes	491 (47%)
No	543 (53%)
* Total years of clinical experience (All)	6.00 ± 5.31
* Year of using telehealth (category)	
<1 yr.	222 (21.47%)
1 to 3 yrs.	150 (14.51%)
4 to 6 yrs.	58 (5.61%)
7 to 9 yrs.	24 (2.32%)
≥ 10 yrs.	37 (3.58%)
**Profession**	
Physician	231 (22.34%)
Nurse	222 (21.47%)
Respiratory therapy	186 (17.99%)
Physiotherapy	120 (11.61%)
Pharmacy	112 (10.83%)
Radiology	29 (2.80%)
Hospital administration	26 (2.51%)
Emergency Medical Services	16 (1.54%)
Occupational therapy	15 (1.45%)
Health education	14 (1.35%)
Operation room technician	13 (1.25%)
Medical Laboratory	8 (0.77%)
Speech-language therapy	7 (0.68%)
Anaesthesia	7 (0.68%)
Public health	6 (0.58%)
Biomedical Engineering	4 (0.38%)
Dietitian	3 (0.29%)
Dentist	3 (0.29%)
Profession not declared	12 (1.16%)

Data are presented as frequencies and percentages. Continuous variables are presented as mean (±SD) or median [IQR]. * Data are reported out of 491 respondents who used telehealth.

**Table 2:** Perception of Telehealth Applications among Users (*n* = 491).

Domains and Items	Strongly Agree	Agree	Neutral	Disagree	Strongly Disagree
**Awareness domain**					
*Telehealth is a useful application in healthcare quality and delivery*	181 (43.30%)	172 (41.15%)	53 (12.68%)	12 (2.87 %)	0 (0.0%)
*Telehealth is easy to use*	167 (39.95.2%)	193 (46.17%)	44 (10.53%)	13 (3.11%)	1 (0.24%)
*I think positively about using telehealth in clinical facilities*	171 (40.91%)	186 (44.50%)	47 (11.24%)	11 (2.63%)	3 (0.72%)
**Attitude domain**					
*I intend to use telehealth when applicable*	164 (39.23%)	183 (43.78%)	57 (13.64%)	11 (2.63%)	3 (0.72%)
*I feel comfortable with information and communication technologies*	180 (43.06%)	172 (41.15%)	53 (12.68%)	11 (2.63%)	2 (0.48%)
*I would recommend the use of telehealth to a patient*	164 (39.23%)	185 (44.26%)	52 (12.44%)	14 (3.35%)	3 (0.72%)
**Practice domain**					
*The use of telehealth changed my working routine*	141 (33.73%)	177 (42.34%)	71 (16.99%)	25(5.98%)	4 (0.96%)
*The use of telehealth enables me to have quicker access to patient information*	159 (38.04%)	172 (41.15%)	69 (16.51%)	16(3.83%)	2 (0.48%)
*Everyone in my workplace uses telehealth*	118 (28.23%)	110 (26.32%)	101 (24.16%)	77(18.42%)	12 (2.87%)
*Telehealth is useful for patients with transportation difficulties*	190 (45.45%)	167 (39.95%)	53 (12.68%)	6 (1.44%)	2 (0.48%)
*Based on my experience, patients accept telehealth*	113 (27.03%)	167 (39.95%)	111 (26.56%)	22 (5.26%)	5 (1.20%)
*Based on my experience, patients like using telehealth*	101 (24.16%)	161 (38.52%)	126 (30.14%)	22 (5.26%)	8 (1.91%)
*If I were a patient, I would like to use telehealth*	149 (35.65%)	162 (38.76%)	72 (17.22%)	28 (6.70%)	7 (1.67%)
**Beliefs domain**					
*I trust that patients are using telehealth equipment effectively*	121 (28.95%)	183 (43.78%)	85 (20.33%)	27 (6.46%)	2 (0.48%)
*I trust that transmitted patients’ information via telehealth is reliable*	142 (33.97%)	193 (46.17%)	63 (15.07%)	19 (4.55%)	1 (0.24%)
*Telehealth ensures the confidentiality of my patients’ information*	160 (38.28%)	194 (46.41%)	49 (11.72%)	13 (3.11%)	2 (0.48%)
*Telehealth is an acceptable method to monitor specific patient outcomes*	138 (33.01%)	187 (44.74%)	73 (17.46%)	17 (4.07%)	3 (0.72%)
*I believe telehealth is a good facilitator to provide effective patient care*	129 (30.86%)	198 (47.37%)	67 (16.03%)	20 (4.78%)	4 (0.96%)
*I can follow-up (reassess) a patient using telehealth*	151 (36.12%)	184 (44.02%)	67 (16.03%)	15 (3.59%)	1 (0.24%)
*I know when to stop using telehealth with a patient*	156 (37.32%)	186 (44.50%)	61 (14.59%)	13 (3.11%)	2 (0.48%)
*Telehealth improves my practice*	137 (32.78%)	159 (38.04%)	97 (4.78%)	20 (4.78%)	5 (1.20%)
*The use of telehealth helps me monitor my patients more rapidly*	140 (33.49%)	181 (43.30%)	71 (16.99%)	23 (5.50%)	3 (0.72%)
*The use of telehealth implied major modifications in my clinical practice*	124 (29.67%)	202 (48.33%)	70 (16.75%)	17 (4.07%)	5 (1.20%)
*My workplace has sufficient technology and structure to support telehealth*	106 (25.36%)	178 (42.58%)	92 (22.01%)	37 (8.85%)	5 (1.20%)
*I think it is a good idea to use telehealth to monitor my patients*	145 (34.69%)	178 (42.58%)	67 (16.03%)	27 (6.46%)	1 (0.24%)
**Training domain**					
*I would like to receive more training on telehealth*	153 (36.60%)	181 (43.30%)	65 (15.55%)	17 (4.07%)	2 (0.48%)
*I feel like I have been sufficiently trained to use telehealth effectively*	124 (29.67%)	169 (40.43%)	82 (19.62%)	38 (9.09%)	5 (1.20%)
*I feel comfortable training patients on how to use telehealth systems independently*	132 (31.58%)	188 (44.98%)	68 (16.27%)	27 (6.46%)	3 (0.72%)
*I feel comfortable to train peers and colleagues on using telehealth*	149 (35.65%)	175 (41.87%)	66 (15.79%)	24 (5.74%)	4 (0.96%)

**Table 3:** Telehealth Use Experience and Rate per Profession in Saudi Arabia (*n* = 491).

Profession	Experience of TH	Clinical Experience	TH Use Rate
Speech-language therapy	4.57 ± 2.43	4.46 ± 2.07	98%
Public health	1.16 ± 0.40	1.10 ± 0.78	95%
Health education	1.85 ± 1.61	1.62 ± 2.52	89%
Emergency medical services	4.00 ± 2.44	4.61 ± 4.38	87%
Radiology	4.10 ± 2.17	3.93 ± 3.23	83%
Biomedical engineering	2.50 ± 2.38	1.25 ± 0.25	82%
Operation room technician	5.53 ± 0.31	7.15 ± 1.99	77%
Respiratory therapy	4.40 ± 2.21	3.35 ± 3.00	76%
Nurse	4.57 ± 1.96	6.14 ± 5.23	74%
Anaesthesia	5.85 ± 0.37	8.14 ± 4.18	72%
Physiotherapy	4.19 ± 2.11	6.05 ± 4.32	69%
Dietitian	4.00 ± 2.64	2.66 ± 2.51	67%
Medical laboratory	2.12 ± 1.72	3.31 ± 2.34	64%
Pharmacy	3.90 ± 2.17	6.33 ± 5.23	62%
Hospital administration	5.15 ± 1.64	9.01 ± 7.20	57%
Occupational therapy	4.66 ± 2.02	8.20 ± 5.17	57%
Profession not declared	4.83 ± 1.80	8.5 ± 6.30	57%
Dentist	1.66 ± 0.57	3.00 ± 0.23	55%
Physician	3.43 ± 2.22	7.67 ± 6.50	44%

**Note:** TH, telehealth. Data are presented as mean and standard deviation. Use rate is presented as a proportion.

## Data Availability

The data underpinning the results reported in this article will be available to researchers who make a methodologically sound proposal within 2 years following article publication. To submit a proposal, please contact s.alghamdi18@imperial.ac.uk.

## Supplementary Materials

The following supporting information can be downloaded at: https://www.mdpi.com/article/10.3390/healthcare10081527/s1, Figure S1: Common barriers of using telehealth applications per profession among non-users; Figure S2: Common barriers among those who used telehealth per profession.
