# Supplementary material for: Healthcare Providers’ Perception and Barriers Concerning the Use of Telehealth Applications in Saudi Arabia: A Cross-Sectional Study
Source: Healthcare (Basel). 2022 Aug 13;10(8):1527. doi: 10.3390/healthcare10081527 (PMC9408269; doi:10.3390/healthcare10081527)

Figure S1: Common Barriers of using Telehealth Applications per Profession among Non-users.

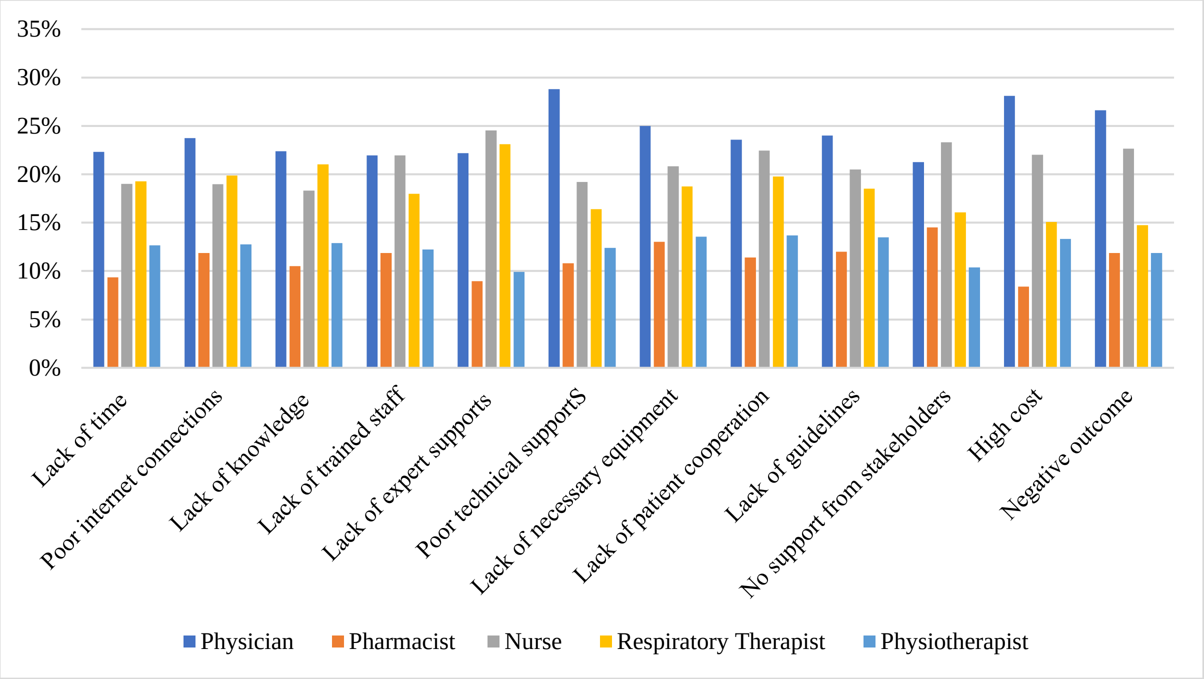

Figure S2: Common Barriers Among those who Used Telehealth per Profession.

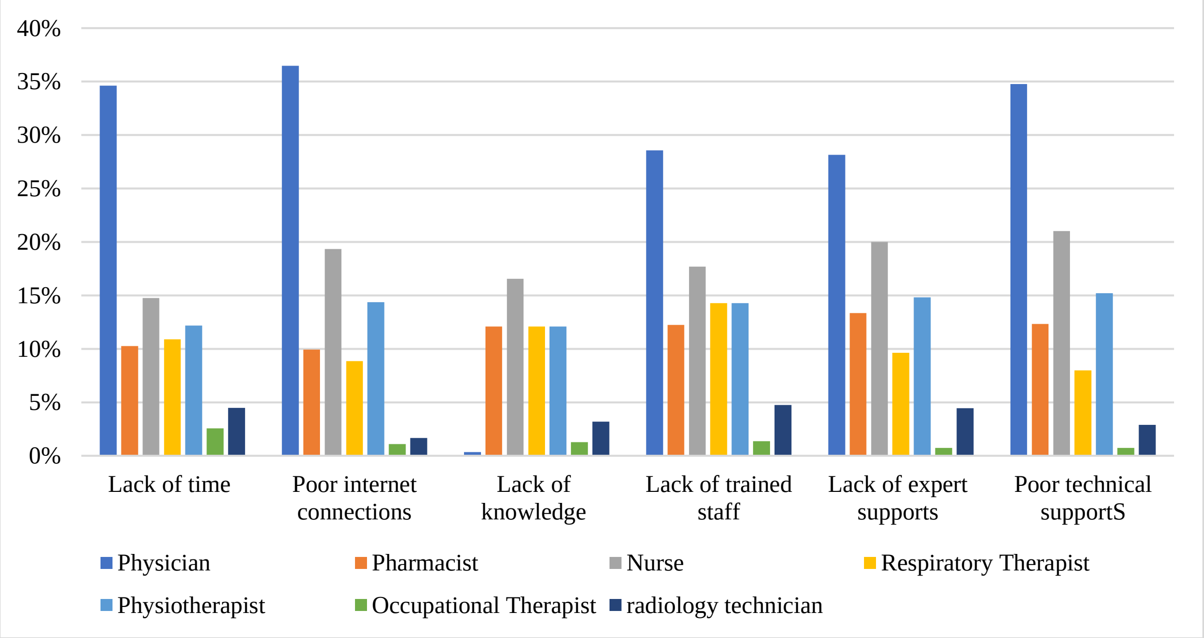

Supplement: Supplementary file 1 [file healthcare-10-01527-s001.zip › healthcare-1806755-supplementary.pdf]
